# Sex Mysteries of the Fly Courtship Master Regulator Fruitless

**DOI:** 10.3389/fnbeh.2019.00245

**Published:** 2019-10-18

**Authors:** Kosei Sato, Junpei Goto, Daisuke Yamamoto

**Affiliations:** ^1^Neuro-Network Evolution Project, Advanced ICT Research Institute, National Institute of Information and Communications Technology, Kobe, Japan; ^2^Division of Neurogenetics, Tohoku University Graduate School of Life Sciences, Sendai, Japan

**Keywords:** *Drosophila*, sexually dimorphic circuit, social effect, mating behavior, transcription factors

## Abstract

The *fruitless* (*fru*) gene of *Drosophila melanogaster* generates two groups of protein products, the male-specific FruM proteins and non-sex-specific FruCOM proteins. The FruM proteins have a 101 amino acids (a.a.)-long extension at the N-terminus which is absent from FruCOM. We suggest that this N-terminal extension might confer male-specific roles on FruM interaction partner proteins such as Lola, which otherwise operates as a transcription factor common to both sexes. FruM-expressing neurons are known to connect with other neurons to form a sexually dimorphic circuit for male mating behavior. We propose that FruM proteins expressed in two synaptic partners specify, at the transcriptional level, signaling pathways through which select pre- and post-synaptic partners communicate, and thereby pleiotropic ligand-receptor pairs for cell-cell interactions acquire the high specificity for mutual connections between two FruM-positive cells. We further discuss the possibility that synaptic connections made by FruM-positive neurons are regulated by neural activities, which in turn upregulate Fru expression in active cells, resulting in feedforward enhancement of courtship activities of the male fly.

## Preface

*fruitless* (*fru*) mutant males in *Drosophila* are known to exhibit strong male-to-male courtship activities with reduced or no female-directed courtship (Hall, 1978; Villella et al., 1997; Yamamoto and Koganezawa, 2013). The gene responsible for *fru* mutant phenotypes encodes, when wild type, a group of transcriptional regulators with a masculinizer function FruM (Ito et al., 1996; Ryner et al., 1996), which organize, together with the other sex-determinant protein Doublesex (Dsx), a subset of neurons into the sexually dimorphic neural circuitry for mating behavior (Kimura et al., 2005, 2008; Cachero et al., 2010; Rideout et al., 2010; Robinett et al., 2010; Ruta et al., 2010; Yu et al., 2010; Kohl et al., 2013; Tanaka et al., 2017). However, there remain uncertainties regarding the mechanisms of action of the *fru* gene in achieving this organizer role in the sexual dimorphism formation of the brain. This article discusses three major questions. Do non-sex-specific products (FruCOM) of the *fru* gene have nothing to do with sex-type specification? Is the neural masculinizing action of FruM ascribable entirely to its cell autonomous function? Does the *fru* gene affect adult behavior exclusively through its developmental functions before adult emergence? In this article, we discuss the importance of the finding that nearly all neuroblasts in both FruM-positive and FruM-negative lineages express FruCOM, the finding that postsynaptic tissues form through interactions with a *fru*-positive presynaptic neuron (non-cell autonomy), and the finding that the *fru*-positive circuit appears to accommodate itself to ambient conditions to best tune the male’s behavior from time to time.

## Multifaceted Fru Protein Activities Rely on Complex Splicing

**Figure 1:**
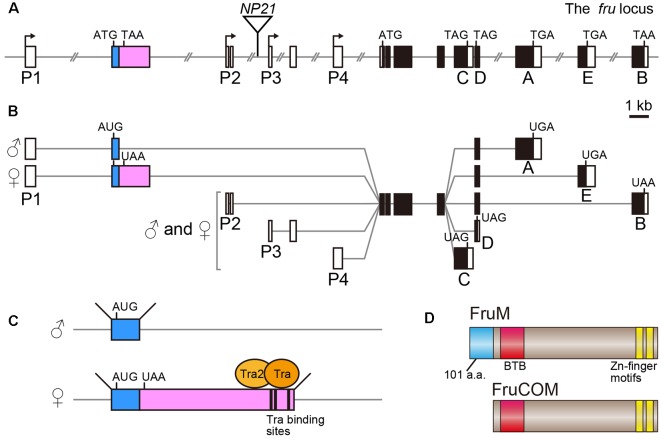
Schematic representation of the *fruitless (fru)* gene structure.** (A)** Locations of four promoters (P1–P4), the exon-intron organization and the *fru*^NP21^ *P*-element insertion point (an inverted triangle) are shown. Filled and open boxes indicate coding and non-coding exons, respectively. The second exon subjected to sex-specific splicing is highlighted in color. A-E denote isoform-specific exons for types A-E. The start and termination codons are also shown. **(B)** Splicing variations and the resulting protein isoform variants are illustrated. **(C)** The Tra-binding sites and sexually dimorphic splicing mechanism are depicted. **(D)** Schematic representation of the FruM and FruCOM protein structures.

The *fru* gene spans over 150 kb of the genome, and harbors at least four promoters, *P1–P4* (Ryner et al., 1996; Usui-Aoki et al., 2000; Figure 1A). The distally located *P1* promoter is dedicated to sex-specific functions of the *fru* gene, whereas the *P2–P4* promoters contribute to the production of FruCOM proteins, which are shared by both sexes (Ryner et al., 1996; Anand et al., 2001; Song et al., 2002; Figures 1B,C). Structurally, FruM proteins have a unique N-terminal extension composed of 101 amino acids (a.a.), followed by the main body of the protein, which is composed of a sequence identical to full-length FruCOM (except for small variations; Ryner et al., 1996; Song et al., 2002; Figure 1D). Thus, although the C-termini are common to FruM and FruCOM, there are five types of C-terminal splice variants called types A to E (Figures 1A,B). For example, the FruM isoform with the C-terminus of type B is referred to as FruBM. Types A, B and E in our terminology (Usui-Aoki et al., 2000) correspond to types A, C and B in the terminology adopted by the Barry Dickson (Demir and Dickson, 2005; Stockinger et al., 2005) and Stephen Goodwin groups (Song et al., 2002). Thus far, the type A, B and E isoforms (following the terminology of Usui-Aoki et al., 2000, which is adopted throughout this article) have been studied in some detail, and so we will focus on these three isoforms in the following discussion. The 101 a.a. extension unique to FruM proteins has no known motif, whereas the main body of the protein has a BTB domain near the N-terminus and two zinc finger motifs at the C-terminus (Ito et al., 1996; Ryner et al., 1996; Figure 1D). The BTB-Zn finger proteins are dominated by transcriptional regulators, and indeed, this proved to be true for FruM as well; FruBM binds to the DNA region named FROS to repress transcription of a target gene (e.g., *robo1*, Ito et al., 2016) that forms a complex with other transcription regulators, including HDAC1, HP1a, Bonus, TRF2 and Lola (Ito et al., 2012; Chowdhury et al., 2017; Sato et al., 2019), some of which are well known for their involvement in chromatin modifications. Although C-terminal variations likely contribute to target specificities (Neville et al., 2014; von Philipsborn et al., 2014), the absence of the male-specific N-terminal extension probably does not narrow the range of target choice, because major portions of the behavioral and cellular phenotypes of FruM-null mutants are rescuable by artificial expression of FruCOM instead of FruM (Ferri et al., 2008). This observation, however, does not exclude the possibility that FruCOM might have additional transcriptional targets to which FruM proteins are unable to bind for transcriptional regulation.

Whereas FruCOM functions as well as FruM in terms of masculinizing neural and behavioral traits, FruCOM and FruM have different endogenous tissue distributions (Lee et al., 2000). The *P1* promoter seems to be active only in neurons, as FruM expression is strictly confined to neurons (Sato et al., 2019). *P1*-derived *fru* mRNAs are transcribed in both females and males (Usui-Aoki et al., 2000), but the FruM protein is male-specific and absent from females (Lee et al., 2000; Usui-Aoki et al., 2000). The male-specific FruM expression is a result of sex-specific splicing of the *fru* primary transcript (Figures 1B,C), which yields *fru* mRNA encoding a full-length ORF in males and an ORF prematurely interrupted by a stop codon (and thus non-coding) in females (Heinrichs et al., 1998). Thus, the presence or absence of FruM (FruCOM is not expressed in adult neurons of either sex) is decisive in directing the sexual fate of a neuron to the male fate or female fate.

The sex-determination in *Drosophila* is achieved on a cell-by-cell basis, i.e., each cell composing the entire organism establishes its sexual identity according to the genetic code without any involvement of sex hormone signaling. When the ratio (X/A) of the number of X-chromosomes over the number of autosome pairs (typically “2”) is 1.0 (such as when somatic cells in an individual carry two X chromosomes) or larger, the cell adopts the female fate, whereas, when the X/A value is 0.5 (in an individual carrying a single X chromosome) or smaller, the cell adopts the male fate. Counting of the relative numbers of X-chromosomes is performed by a transcriptional two-directional switch at the *Sex-lethal* (*Sxl*) gene, which is transcribed only when X/A exceeds 1.0. Thus the *Sxl* gene typically produces the Sxl protein only in XX individuals. The female-specific Sxl protein functions as a splicing regulator that induces female-specific splicing of its target, the *transformer* (*tra*) gene primary transcript. Only a transcript spliced in the female pattern can encode a functional Tra protein, which in turn induces female-specific splicing distinct from a default splicing that occurs in males in its targets, e.g., the primary transcript from the *P1* promoter of the *fru* gene (*fru-P1*). Upon binding to the Tra target motif in the *fru-P1* primary transcript (Ito et al., 1996; Ryner et al., 1996; Heinrichs et al., 1998), the Tra protein induces splicing of the *fru-P1* primary transcript at the site 3′ to the binding site in females, leading to the production of an mRNA whose ORF is interrupted by a termination signal (Ito et al., 1996; Ryner et al., 1996; Heinrichs et al., 1998; Figure 1C). In males, default splicing in the absence of Tra takes place at a more 5′ site, which excludes the termination signal from the mature *fru* mRNA (Ito et al., 1996; Ryner et al., 1996; Heinrichs et al., 1998). *fru* is therefore considered to be an effector transcription factor gene in the sex determination cascade, together with the other Tra target, *dsx*.

## Does Male-Specific FruM Signaling Intersect Non-Sex-Specific Frucom Signaling?

No Tra-binding motif has been identified in primary transcripts from *P2–P4* promoters. The *P1* promoter dedicated to sex-related functions is active only in neurons, while the *P2–P4* promoters are active in a variety of tissues. The apparent absence of FruCOM (*P2–P4* products) in neurons and neuron-restricted FruM (*P1* products) expression do not necessarily mean that FruCOM is “non-neural.” Lee et al. (2000) observed a large number of cells labeled by the anti-FruCOM but not anti-FruM antibodies in the brain and ventral nerve cord of third instar female and male larvae. Our recent analysis with wandering stage larval brains convincingly showed that nearly all neuroblasts transiently express FruCOM proteins, which rapidly fade out and disappear in the daughter cells (i.e., ganglion mother cells and neurons; Sato et al., 2019). This raises the intriguing possibility that FruCOM proteins have a hitherto uncharacterized function in proliferating neuroblasts, such as specifying the types of neurons the neuroblast should produce. A comprehensive analysis of clonal cell lineages unraveled that *P1*-dependent *fru*-positive neurons (hereinafter *fru*[+]-neurons) that are sexually dimorphic derive from multiple neuroblasts rather than a few dedicated neuroblasts: in fact, all type II neuroblast lineages bring about sexually dimorphic *fru*[+]-neurons (Ren et al., 2016). It remains to be determined whether larval expression of FruCOM could have any sustained effect on the transcriptional state of Fru-responsive genomic elements—such as, for example, to sensitize them for subsequent exposure to FruM.

## Do FruM Proteins Shape Only Neurons in Which They Are Expressed?

With a few exceptions, transcription factors act within cells in which they are expressed. Indeed, the FruM proteins, in their capacity as transcription factors, specify the structure of a *fru*[+]-neuron by their cell autonomous functions. The best characterized *fru*[+] neurons are those that compose the mAL cluster in the brain. mAL neurons are sexually dimorphic in three respects (Kimura et al., 2005). First, the number of neurons that comprise the cluster is five in females and 30 in males. Second, the ipsilateral neurite forms only in males. Third, the posteriorly extending contralateral neurite bifurcates near its tip only in females. These three sex-specific characteristics are determined by the presence or absence of FruM. Reducing functional FruM levels in males (e.g., in *fru* hypomorphic mutant males) leads to an increase in the proportion of female-typical neurons at the expense of the male-type neurons in the mAL cluster (Ito et al., 2012). In principle, the neurons with intersexual structures are not produced; every neuron in the mAL cluster is either a perfect female-type or male-type neuron under *fru* loss-of-function conditions (Ito et al., 2012). By contrast, manipulations of a *fru* downstream element or some *fru*-interacting partners result in malformation of one or more sexually dimorphic characteristics of mAL neurons (Goto et al., 2011; Ito et al., 2016; Chowdhury et al., 2017; Sato et al., 2019). These observations suggest that FruM proteins operate as two-directional switches between the female-type and male-type developmental pathways in mAL neurons, whereas the specification of each sex-specific neural structure is achieved by pathway-specific molecules downstream of FruM. FruM and the FruM-downstream components function in the cell that produces these molecules, i.e., they function cell autonomously in conferring the sex-specific characteristics onto mAL neurons. Sex differences in neurons other than mAL are also produced by a similar cell autonomous mechanism, and sexually dimorphic neurons thus specified on a cell-by-cell basis may form synapses to establish a sex-specific circuitry. On the other hand, synaptogenesis inevitably involves coordinated tuning of pre- and postsynaptic elements. Thus, it is conceivable that cell-to-cell interactions during synaptogenesis would also contribute to sexually dimorphic refinement of dendritic arbors and axonal terminals. There is a precedent case in which FruM expression in a cell was shown to be pivotal for normal development of another cell; that is, the male-specific adult muscle called the muscle of Lawrence (MOL) was shown to form only when innervated by a male motoneuron named the Mind (MOL-inducing) neuron (Nojima et al., 2010), irrespective of whether the muscle on its own is composed of female cells or male cells (Lawrence and Johnston, 1984, 1986; Taylor, 1992). Muscle cells do not express FruM and the MOL is not an exception to this rule. Exploring how the MOL induction is achieved by the Mind neuron will provide insights into the molecular mechanism whereby FruM in a neuron exerts non-cell autonomous effects on its synaptic partners for the formation of a sexually dimorphic circuit.

## Do FruM Function Only in Development Or Do FruM Also Function in a Behaving Adult Fly?

The nervous system of holometabolous insects such as *Drosophila* is largely reorganized during the pupal stage when sexually dimorphic circuitry is newly established under the control of FruM and Dsx. Consistent with this fact, FruM expression commences at the wandering third instar larval stage, peaks at the pupal stage, and thereafter declines but does not disappear after the adult emergence (Lee et al., 2000). The functions of FruM in the adult stage have been ill-defined. However, clues to the roles of FruM in adults were obtained by Hueston et al. (2016). They found that *fru-GAL4* expression in the *Or47b*-expressing olfactory neurons is sustained through the adult stage only when these cells are functionally active: *fru-GAL4* expression is activity-dependent in *Or47b* neurons (Hueston et al., 2016). Or47b is activated by the fatty acid ligand methyl laurate, which is an endogenous aphrodisiac for both sexes and is contained in the adult cuticle of both sexes (Dweck et al., 2015). The major sex pheromones in *Drosophila* are several hydrocarbon compounds in the body surface cuticle (Jallon, 1984). Notably, genetic deprivation of all hydrocarbons from wild-type male flies makes them extremely attractive for other males and results in male-male courtship, which is rarely seen under normal conditions (Billeter et al., 2009). These unusual homosexual activities among males are likely evoked by the fatty acid attractants remaining in the cuticle, from which hydrocarbon pheromones, both excitatory and inhibitory ones, have been deprived. Notably, male-male courtship is a hallmark of *fru* mutants that lack *fru* expression (Hall, 1978; Villella et al., 1997). Recent studies have demonstrated that male-male courtship in *fru* mutants is enhanced by rearing these flies in a group and suppressed by social isolation (Pan and Baker, 2014; Kohatsu and Yamamoto, 2015). Olfactory experience appears important for the development of this trait because genetic deprivation of olfaction abrogated the induction of male-male courtship in grouped *fru* mutant males (Pan and Baker, 2014). These observations tempt us to postulate that activity-dependent *fru* expression might play a role in experience-dependent changes in behavior after adult emergence. Another study showed that juvenile hormone (JH; known to stimulate reproductive maturation in the adult) acts on *Or47b* olfactory neurons in mature adult males to boost their ligand sensitivity, making these elder males more successful in copulation than younger males (Lin et al., 2016). This finding invites speculation that JH might act through FruM to elevate Or47b sensitivity. Remarkably, Wu et al. (2018) suggested that some of the JH actions are mediated by a FruM-dependent mechanism: they showed that a sex difference in sleep patterns disappears and FruM expression in the brain declines in male flies when JH signaling is inhibited. Of note, sleep activities and sexual activities are reciprocally regulated by a group of *fru*[+] neurons called P1 neurons (Chen et al., 2017), which were originally identified to be the primary decision-making cells for the initiation of male courtship (Kimura et al., 2008). It would be of interest to examine whether the mechanism by which JH elevates male mating success by acting on Or47b is dependent on functional FruM in these neurons. A recent study revealed that *IR52a*-expressing *fru*[+]-chemosensory neurons on the wing margin mediate input to stimulate male-male courtship (He et al., 2019). It remains to be examined whether *fru* expression in the *IR52a*-sensory neurons as positive regulators for male-male courtship is also modulated by neural activities during the adult stage.

## Perspectives

The *fru* gene produces two major protein groups: FruM and FruCOM. The FruM proteins have an N-terminal extension that FruCOM proteins lack, but we do not know how important this structural difference is in terms of the protein functions. The expressions of the FruM and FruCOM proteins are mutually exclusive both spatially and temporally (e.g., neuroblasts vs. neurons in the postembryonic nervous system; Sato et al., 2019), implying that each protein group acts in a different developmental context, possibly through partially redundant signaling mechanisms.

Molecular studies on the actions of FruBM protein have revealed that this protein forms a transcriptional complex with an isoform of Lola, a pleiotropic transcription factor, to transcriptionally repress the *robo1* gene, a direct target of FruM (Ito et al., 2016). In male flies, FruM protects Lola from truncation upon binding to Lola through each-others’ BTB domains; the N-terminal portion of Lola is otherwise truncated by ubiquitin proteasome digestion (Sato et al., 2019). Robo1 functions to inhibit the extension of the male-specific neurite of mAL neurons, thereby contributing to the formation of sexual dimorphism in these neurons (Ito et al., 2016). Full-length Lola represses *robo1* in males, whereas truncated Lola inhibits full-length Lola’s action to repress *robo1*, with the result that the ipsilateral neurite forms in males but not females (Sato et al., 2019). Lola is known to drive neuroblasts to exit the stem cell state and enter the differentiation pathway (Southall et al., 2014). An intriguing possibility is that FruCOM contributes to this process together with Lola in both sexes by playing a transcriptional role similar to that of FruM in sexual-type specification in males, and yet its target specificity or its preference for interaction partners differs from that of FruM. Notably, fasciculation and path-finding of pioneering axons in the embryo were disrupted by *fru* mutations that lost FruCOM while retaining FruM proteins (Song et al., 2002). In the embryonic nervous system, FruCOM but not FruM proteins are expressed in neuroblasts, ganglion mother cells (GMCs) and some neurons and glial cells (Song et al., 2002). Remarkably, axon guidance and fasciculation defects were rescued by the type A or type B isoform of FruCOM (but not by any of the FruM isoforms) when these proteins were overexpressed in neuroblasts and GMCs (but not neurons), suggesting that FruCOM functions are required in cells before neural differentiation for normal axonogenesis that occurs after differentiation (Song et al., 2002). Intriguingly, FruM overexpression even exaggerated the axonal defects in *fru* mutants (Song et al., 2002). These observations imply that FruCOM proteins with no N-terminal extension have biological activities distinct from those of FruM with the N-terminal extension. One may envisage, for example, that the male-specific N-terminal extension of FruM affects the stability of the FruM-containing transcriptional complex by modulating the proteasomal degradation of FruM-interaction partners within the complex. We presume that FruM is evolutionarily a derivative of FruCOM that was co-opted for sex-specific functions in neurons, whereas FruCOM expression was eliminated through negative selection in evolution.

The *robo1* gene is the sole established target of FruM (more specifically, FruBM; Ito et al., 2016), although the total number of FruBM targets is expected to exceed 100 based on immunolabeling of FruBM that bound to the target sites on polytene chromosomes (Ito et al., 2012). Robo1 is a transmembrane protein that functions as a receptor for Slit proteins, membrane-anchored ligands that mediate cell-to-cell interactions (Kidd et al., 1998, 1999). Robo proteins of vertebrates and invertebrates exert pleiotropy, working in neural midline crossing/turning/stopping, angiogenesis, kidney development, heart development, mammary gland morphogenesis and other developmental processes, and this pleiotropy partly depends on the pleiotropic processing of Robo and Slit upon their binding to each other (Blockus and Chédotal, 2016), which occurs in two facing cells that interact with each other. This leads to an important question. How do FruM-expressing neurons recognize each other and specifically make connections with an appropriate partner? An intriguing possibility is that FruM proteins determine, at the transcriptional level, the manner of processing of Robo and Slit upon ligand-receptor interactions. One can anticipate that only FruM-expressing cells display coherent processing patterns in both pre- and postsynaptic membranes, allowing stable connections to be made and inductive interactions to occur between them.

The loss of FruM expression by the olfactory receptor mutations observed in adult pheromone neurons (Hueston et al., 2016) might suggest that functional synaptic connections are maintained by FruM, whose expression is maintained in a use-dependent manner: a feedforward loop between the neural activity and FruM expression could operate to enhance courtship activities for improved fitness of elder males.

These considerations prompt us to speculate that the *fru* gene became potentiated to achieve a specialist role—i.e., a neural masculinizer role—by creating structurally distinct FruM proteins in addition to FruCOM proteins. We assume that FruM proteins specify coherent signaling pathways in the pre- and postsynaptic neuron pair to form a Fru-labeled neural circuit. This circuit is probably consolidated by the fly’s experience *via* use-dependent synaptic enhancement. However, this model describing how the actions of *fru* could induce adaptive changes in the nervous system of a fly during its individual lifetime remains to be tested in future experiments.

## Author Contributions

DY: conceptualization, review and editing. KS and DY: funding acquisition and writing the original draft. JG: experimental work. KS: result analysis and visualization.

## Conflict of Interest

The authors declare that the research was conducted in the absence of any commercial or financial relationships that could be construed as a potential conflict of interest.
